# A mouse model replicating hippocampal sparing cranial irradiation in humans: A tool for identifying new strategies to limit neurocognitive decline

**DOI:** 10.1038/srep14384

**Published:** 2015-09-24

**Authors:** Wolfgang A. Tomé, Şölen Gökhan, N. Patrik Brodin, Maria E. Gulinello, John Heard, Mark F. Mehler, Chandan Guha

**Affiliations:** 1 Institute for Onco-Physics, Albert Einstein College of Medicine, Bronx, NY 10461, USA; 2 Department of Radiation Oncology, Montefiore Medical Center, Bronx, NY 10461, USA; 3 Department of Neurology, Albert Einstein College of Medicine, Bronx, NY 10461, USA; 4 Department of Neuroscience, Albert Einstein College of Medicine, Bronx, NY 10461, USA; 5 Department of Psychiatry and Behavioral Sciences, Albert Einstein College of Medicine, Bronx, NY 10461, USA

## Abstract

Cancer patients undergoing cranial irradiation are at risk of developing neurocognitive impairments. Recent evidence suggests that radiation-induced injury to the hippocampi could play an important role in this cognitive decline. As a tool for studying the mechanisms of hippocampal-dependent cognitive decline, we developed a mouse model replicating the results of the recent clinical RTOG 0933 study of hippocampal sparing whole-brain irradiation. We irradiated 16-week-old female C57BL/6J mice to a single dose of 10 Gy using either whole-brain irradiation (WBRT) or hippocampal sparing irradiation (HSI). These animals, as well as sham-irradiated controls, were subjected to behavioral/cognitive assessments distinguishing between hippocampal-dependent and hippocampal-independent functions. Irradiation was well tolerated by all animals and only limited cell death of proliferating cells was found within the generative zones. Animals exposed to WBRT showed significant deficits compared to sham-irradiated controls in the hippocampal-dependent behavioral task. In contrast, HSI mice did not perform significantly different from sham-irradiated mice (control group) and performed significantly better when compared to WBRT mice. This is consistent with the results from the RTOG 0933 clinical trial, and as such this animal model could prove a helpful tool for exploring new strategies for mitigating cognitive decline in cancer patients receiving cranial irradiation.

Recent clinical and preclinical investigations have suggested that radiation-induced hippocampal injury could play a major role in the ensuing cognitive decline of patients undergoing whole-brain or partial cranial irradiation^1^,^2^,^3^,^4^,^5^. Hippocampal-related cognitive decline is thought to specifically lead to deficits in memory, learning and spatial processing. Technical advances within the field of clinical radiation therapy have made it possible to physically limit the radiation dose delivered to the hippocampus in whole-brain or partial cranial irradiation^3^,^4^,^6^. Importantly, a recent single-arm prospective phase II study (RTOG 0933) showed a significant reduction in cognitive decline in adult patients with brain metastases treated with hippocampal sparing whole-brain radiation therapy (HS-WBRT), as compared to historical controls treated with conventional WBRT^7^. One of the main hypotheses for sparing the hippocampus from irradiation is protecting the neural stem cell (NSC) compartment in the subgranular zone of the dentate gyrus in the hippocampus. In humans it has been shown that constitutive neurogenesis (the generation of new neurons) occurs within the hippocampus throughout adulthood^8^. In rodents it has been demonstrated that this adult NSC compartment is vital for the *de novo* generation of neurons involved in memory functions^9^,^10^,^11^. Despite recent evidence that protecting the hippocampi during radiation therapy might be important, the mechanisms underlying radiation-induced hippocampal injury are still not well understood. Several possible explanations include suppression of NSC-mediated neurogenesis, death of existing NSCs from radiation or promotion of other neural lineages under the influence of inflammatory cytokines produced by radiation^12^. As such, physical shielding of the hippocampi represents but one of many possible strategies for limiting neurocognitive decline in patients receiving cranial irradiation. To this end, we aimed to develop a mouse model that could replicate the results shown in the RTOG 0933 hippocampal avoidance study, providing researchers with a tool for developing new clinical mitigation strategies and increasing our understanding of hippocampal radiation injury.

## Methods

### Animals and irradiation procedure

**Figure 1:**
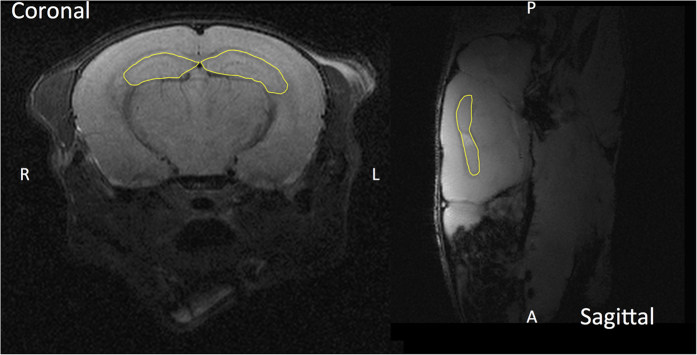
Anatomical outline of the mouse hippocampi. Mouse hippocampi highlighted as a yellow contour on coronal and sagittal T1-weighted magnetic resonance images, obtained using a 9.4 T small animal magnetic resonance imager. The right/left and anterior/posterior directions are indicated in respective panels.

We used 16-week-old female C57BL/6J mice (The Jackson Laboratory, Maine, USA) for all cranial irradiation experiments performed in this study. The irradiation protocols were performed using the image-guided target localization capabilities of the small animal radiation research platform (SARRP, Xstrahl, Surrey, UK). All procedures involving the mice were conducted in accordance with an animal protocol approved by the Institutional Animal Care and Use Committee at the Albert Einstein College of Medicine. To ensure a highly reproducible treatment setup, the mice were anesthetized using a continuous flow of 1.5 liters/minute of 1.5% isoflurane in pure oxygen and immobilized using a custom fixation system prior to radiation delivery. Animals either received whole-brain irradiation or hippocampal sparing irradiation to a dose of 10 Gy delivered in a single fraction, equivalent to a dose of 30 Gy delivered in 2 Gy fractions, assuming an α/β-ratio of 2 Gy. For both irradiation protocols, the cone-beam computed tomography (CBCT) image-guidance of the SARRP was used to determine the lateral irradiation fields, and the dose calculation engine was used to calculate the irradiation time, ensuring efficient, accurate, and reproducible delivery of the intended radiation. The hippocampi are located in the superior half of the mouse brain, as shown in Fig. 1 on a T1-weighted magnetic resonance image.

#### Whole-brain irradiation (WBRT)

**Figure 2:**
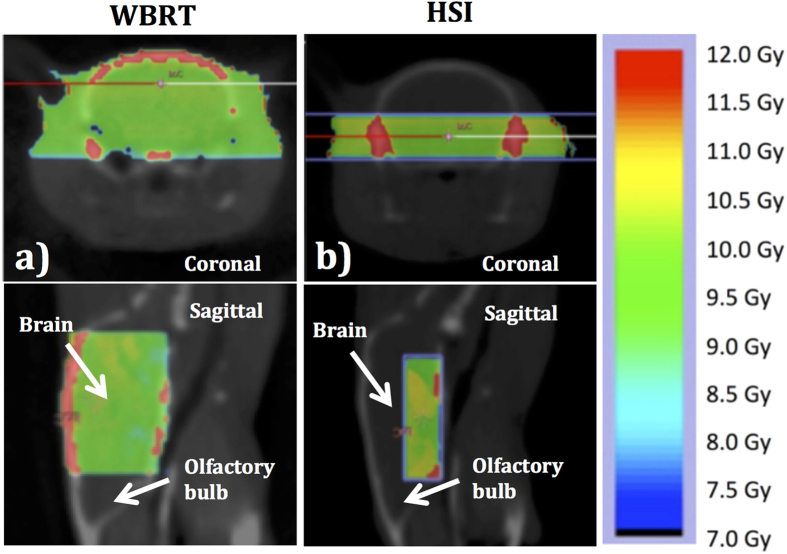
The calculated radiation dose distribution for WBRT and HSI. The calculated radiation dose is shown as a dose color-wash for (**a**) whole-brain irradiation (WBRT) and (**b**) hippocampal sparing irradiation (HSI), with the color bar on the right hand side showing the corresponding dose levels.

Whole-brain irradiation was delivered using a 10 mm × 10 mm collimator, resulting in an irradiation field covering the entire brain while sparing the olfactory bulbs, as shown in Fig. 2a. Half of the radiation dose was delivered from a 90° angle and the second half of the dose from −90°, to ensure homogeneous radiation delivery, at a dose rate of 2.7 Gy/min with the whole-brain collimator, resulting in a treatment time of less than 240 seconds per mouse.

#### Hippocampal sparing irradiation (HSI)

Hippocampal sparing irradiation was delivered using a 3 mm × 9 mm collimator, which in this case resulted in an irradiation field covering the ventral part of the brain, avoiding the hippocampus and the olfactory bulb, cf. Fig. 2b. Similarly to the whole-brain scenario, the radiation was delivered from 90° and −90° angles, although with the smaller collimator the dose rate was 2.5 Gy/min, resulting in a treatment time of 240 second per mouse.

Animals used as sham-irradiated controls were anesthetized and CBCT imaged the same as the irradiated animals (delivering a negligible radiation exposure of about 0.02–0.03 Gy), but not given any cranial irradiation. Taking into account the time needed for anesthesia, setup, and CBCT imaging, the above irradiation techniques allowed us to treat approximately 5 mice per hour.

### Behavioral testing to assess neurocognitive deficits

The battery of behavioral tests included non-specific tests to determine general cognitive performance, anxiety levels and olfactory abilities of the animals. To assess whether cognitive deficits could be ascribed to hippocampal injury, tests were also included that specifically evaluated tasks dependent on hippocampal function and intact neural stem cell survival, proliferation, lineage elaboration and the integration of downstream progeny into existing neuronal networks. The following behavioral tests were performed for all irradiated and control animals.

#### Open field (day 7 post irradiation)

The open field test was used to explore locomotor activity^13^ as well as anxiety and habituation deficits^14^,^15^. Mice were placed in an opaque Perspex arena (16 inches × 16 inches) and allowed to explore the arena for 9 minutes, during which time locomotion (track length, e.g. total distance traveled) and thigmotaxis (time in the periphery vs. time in the center) were recorded automatically using tracking Software (Viewer: Biobserve, Bonn, Germany).

#### Olfactory test (days 9 and 10 post irradiation)

This was a sensory assay measuring the olfactory ability of the animals on a standard buried food test^16^. Mice were fed peanuts for 7–10 days prior to assessment and then food deprived for 1 day before the test. The test involved placing the animal in the Perspex arena with 5 small containers, filled with clean bedding. A peanut was buried in one of the containers. The latency to dig into the correct container to reveal the hidden food was recorded.

#### Elevated plus maze (EPM) (day 12 post irradiation)

This test essentially determines a preference between a comparatively safe and comfortable environment (the closed arms) and a risky environment (elevated open spaces). The general principle is that the more “anxious” the subjects are, the less likely they are to explore a risky or threatening environment^17^. The EPM has been validated pharmacologically, ethologically and with other tests of anxiety-like behaviors. The animals were placed in one of the closed arms to start. The number of entries into each portion of the EPM (open and closed) was scored in addition to the total time spent in each portion of the maze.

#### Object Placement Test (Days 8 and 11 post irradiation) and Object Recognition Test (Day 15 or 16 post irradiation)

To assess cognitive deficits that are dependent on intact neural stem cell niche and NSC-mediated neurogenesis vs. non-specific cognitive function we chose the object placement (a.k.a. novel object location) task and object recognition task, respectively. Object recognition was performed on day 15/16 post irradiation and object placement on days 8 and day 11 post irradiation, as different retention intervals were used that could not be tested on the same day.

Recognition and spatial memory were tested in the novel object recognition task and object placement task (a.k.a. novel object location task) respectively, following established protocols^14^,^15^,^18^,^19^,^20^,^21^. All objects have been extensively validated to ensure that no intrinsic preference or aversion was observed and animals explored all objects similarly. Exploration of the objects was defined as any physical contact with an object (whisking, sniffing, rearing on or touching the object) or orienting to the object from within 5 cm. Tracking software (Viewer: Biobserve, Bonn, Germany) was used to record the sessions.

**Figure 3:**
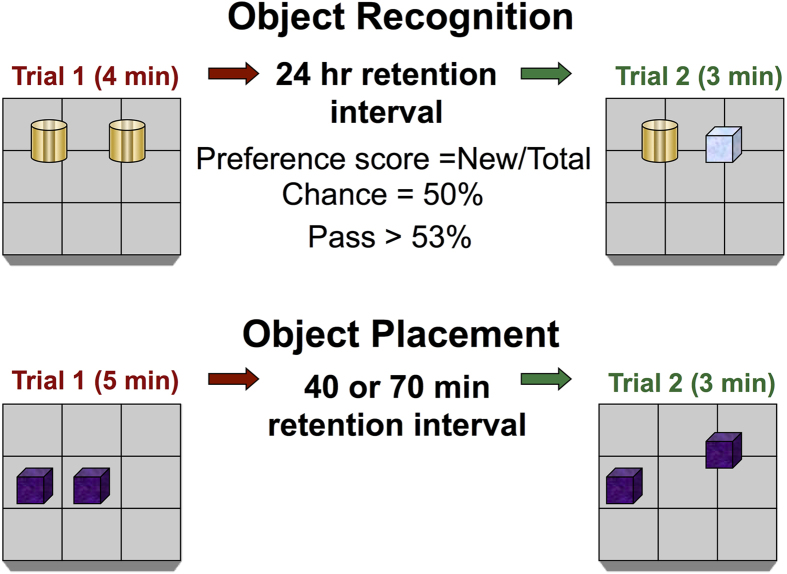
Experimental setup for the object recognition and object placement behavioral assessment tasks. The object recognition task replaces a familiar object with a novel object (top panel) and the object placement task alters the position of one of two familiar objects (lower panel).

In the *object recognition* test, mice were placed in the open field arena and allowed to freely explore two identical objects for 4–5 min. Exploration of each object (in seconds) was recorded for 4 min (trial 1, training). After spending a retention interval of 24 hr in their home cages, the animals were returned to the same arena for 3 min (trial 2, test), now containing one object from trial 1 (familiar object) and one novel object, cf. Fig. 3. The exploration of the novel and familiar objects (in seconds) were recorded.

In *object placement* tests, spatial memory was assessed in a similar way. In trial 1, mice were allowed to explore two identical objects for 5 min in the arena. After spending a retention interval of between 40–70 min in their home cages, mice were returned to the testing arena for 3 min with one object moved to a novel location (trial 2), cf. Fig. 3. Care was taken to ensure that the change of placement alters both the intrinsic relationship between objects and the position relative to internal visual cues.

For both the object placement and object recognition tests, a percentage preference score was derived as 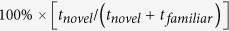, where *t*_*novel*_ is the exploration duration of the novel object and *t*_*familiar*_ is the exploration duration of the familiar object. An exploratory preference score of 50% thus indicates that the subject spent equal times exploring the novel and familiar objects. Results of this test are reported as success rates (pass/fail)—the proportion of animals in each group performing higher than chance (i.e. preferring the novel object). For this purpose, preference scores higher than 53% were defined as “passing”, based on the following rationales: Firstly, during our extensive validation of these tests, we determined that animals with preference scores higher than 53% consistently demonstrate novel object preferences when re-tested, whereas animals with scores lower than 53% consistently show no novel object preference (unpublished data). Secondly, the measure has been previously validated, published and reproduced in several cohorts^18^,^20^,^21^,^22^. It should be noted that animals not exploring the objects in the object placement and recognition tests for at least 3 seconds were excluded from further analyses.

### Alterations in neural stem cell (NSC) proliferation, neurogenesis and inflammation

To confirm the findings from behavioral assessment, we also investigated NSC survival, proliferation and lineage commitment as a result of cranial irradiation. Firstly, using immunohistochemistry we examined cell death within different areas of the brain 6 hours after delivering a single dose of 10, 12, 15 or 20 Gy to the whole brain excluding the olfactory bulbs. This was done by TUNEL assay and combined with DAPI staining to show the number and localization of TUNEL+ apoptotic cells.

Secondly, we examined the effects on proliferating cells, neurogenesis and inflammatory microglial cells following either WBRT or HSI with 10 Gy. This was assessed by doublecortin (DCX) staining to detect neurons, BrdU staining for proliferating cells and Iba-1 to stain for microglia, at 48 hours and 8 days following radiation exposure.

### Tissue processing and immunohistochemistry

Mice were anesthetized with Ketamine and Xylazine according to the guidelines of the Institutional Animal Care and Use Committee at the Albert Einstein College of Medicine for the harvesting of their brains. We utilized intracardiac perfusion of 15 ml of cold PBS containing 2% Heparin, followed by 35 ml of 4% paraformaldehyde (PFA), pH 7.4, to fix the brains. Subsequently brains were removed and embedded in OCT and cut into 30 μm sections before being processed for immunohistochemistry (IHC). Six consecutive hippocampal sections were collected from 6 animal brains to manually quantify immunoreactive cells. Images were captured by an Olympus Bx51 fluorescent microscope with Olympus MicroSuite^TM^.

### Antibody dilution and isotype for IHC

BrdU, rat IgG (1:100, Abcam); Iba-1, polyclonal rabbit IgG (1:700, Wako Chemicals, USA), Doublecortin (DCX) goat IgG (1:400, Santa Cruz). Secondary antibodies used were conjugated to Alexa-fluor-488, Alexa fluor-594 or Alexa fluor-647 (1:1500) (Molecular Probes, Invitrogen, Carlsbad, CA, USA).

### BrdU incorporation study

All mice were injected intraperitoneally with 100 μl of 10 mg/ml BrdU in 0.9% NaCl, 7 mM NaOH 90 minutes before irradiation. For IHC, brain sections were incubated at 65 °C for 30 minutes in 50% Formamide in 2X SSC, rinsed with PBS for 5 minutes before being treated with 2 N HCl solution 37 °C for 30 minutes and neutralized with 0.1 M sodium borate solution, pH 8.5, for 10 min, washed with PBS for 3 minutes, then quenched with 0.1% sodium borohydride for 5 min at room temperature before a final wash with PBS 0.1% Tx100 and then incubated sequentially with anti-BrdU antibody and secondary antibody against anti-BrdU. Thereafter, the tissues were subjected to IHC for DCX and Iba-1 as previously described elsewhere^23^.

### Statistical analysis

The results of all behavioral assessment tests were analyzed using JMP (SAS, Cary, NC), and for each test the different treatment groups were compared using a likelihood ratio test for χ^2^ distributions. If likelihood ratio tests showed a significant difference between groups (p < 0.05), we performed pairwise χ^2^ comparisons to analyze which groups exhibited significantly different performance profiles.

The IHC data were analyzed using a one-way analysis of variance (ANOVA) test to determine whether groups were significantly different. This was then followed by *post hoc* Tukey’s multiple comparisons tests to determine which groups were significantly different. Thus, 3 statistical comparisons were performed per data set and to reduce the false discovery rate we applied the Benjamini-Hochberg correction for multiple hypotheses testing^24^. In short, comparisons were arranged in monotonically increasing order according to their p-values from *p*_(1)_ to *p*_(*m*)_. Starting from the largest p-value, *p*_(*m*)_, only those comparisons {1*,…,k*} for which *p*_(*k*)_ satisfies the constraint 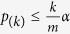 are considered statistically significant, where *m* is the total number of comparisons per data set (3 in this case), and α = 0.05.

## Results

The irradiation procedure was well tolerated and none of the animals showed any physical signs of distress or discomfort following cranial irradiation. Animals were tested in 4 cohorts of 20–24 animals per cohort, with control and WBRT represented in every cohort. There was good reproducibility between cohorts and cohorts were not statistically different from one another, and were thus combined in the analyses. After performing initial testing of object placement at 40 min retention interval it became clear that it would be beneficial to also include longer delay times for this test (70 min retention) and to include object recognition tests as well. As mentioned previously, animals were excluded from the preference score analysis if they did not explore the objects for at least 3 seconds. As such, a total of 67 animals were subjected to the object placement test with 70 min retention interval (10 excluded from analyses), 95 animals were subjected to the object placement with 40 min retention (15 excluded from analyses), and 58 animals were subjected to the object recognition test (5 excluded from analyses). The number of animals excluded was not different between treatment groups and similar to what is typically found (about 10–15%).

We also included more animals in the HSI treatment group as the preliminary behavioral data showed larger variance for this group compared to non-irradiated controls and WBRT animals.

### Behavior in non-hippocampal specific tasks was unaffected by cranial irradiation

**Figure 4:**
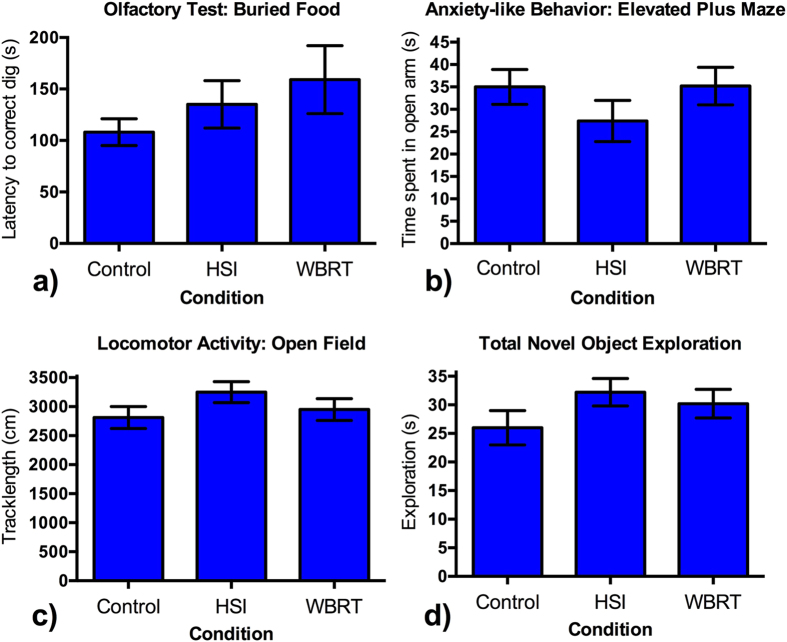
Results from non-hippocampal-dependent behavioral tasks. Panel (**a**) shows the results of the Olfactory Test (buried food); Panel (**b**) shows the results from Elevated Plus Maze (anxiety-like behavior); Panel (**c**) shows Locomotor Activity; and Panel (**d**) shows Total Novel Object Exploration. The data are presented as mean ± 1 standard deviation. There was no significant difference among groups in any of these tests.

The results of non-hippocampal behavioral tasks are presented in Fig. 4. Olfactory ability was similar for all groups in a standard buried food task in which the animals were food deprived for 1 day and the latency to find a peanut hidden under clean bedding in one of 5 cups was measured. Anxiety-like behavior was also similar between groups in the elevated plus maze, in which longer times spent in the open arm indicate less anxiety-like behavior. Locomotor activity, assessed as track length in an open field test, was also not different between groups. General object exploration was also assessed in the training trial of the object placement test and again no difference was detected between groups.

Taken together, these data indicate that exposure to either whole-brain radiation (WBRT) or hippocampal sparing radiation (HSI) did not non-specifically alter the general behavioral profile of the assessed mice.

### Spatial memory deficits are evident after WBRT, but not after HSI

**Figure 5:**
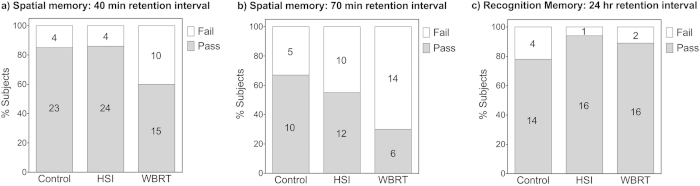
Reduced performance is seen in the highly hippocampal-dependent object placement task after WBRT, but not after HSI. Panel (**a**) shows the results of the object placement task following a 40 min retention interval (OP40) 8 days after irradiation; panel (**b**) shows the results of the object placement task following a 70 min retention interval (OP70) 11 days after irradiation. Panel (**c**) shows the results of the object recognition test following a 24 hour retention interval.

Animals exposed to WBRT showed significant deficits compared to sham-irradiated controls in the object placement task after a 40 min retention interval (χ^2^_df(52,1)_ = 2.14, p = 0.0386) (cf. Fig. 5a) and a 70 min retention interval (χ^2^_df(35,1)_ = 4.73, p = 0.0296) (cf. Fig. 5b). In contrast, HSI mice did not perform significantly different from sham-irradiated mice in either the 40 min (χ^2^_df(55,1)_ = 0.003, p = 0.956) or 70 min (χ^2^_df(37,1)_ = 0.549, p = 0.459) retention intervals and performed significantly better compared to WBRT mice in the 40 min retention interval (χ^2^_df(53,1)_ = 2.29, p = 0.032) (cf. Fig. 5a,b). The same trend was seen in the 70 min data with HSI mice performing better than WBRT mice, although this did not reach statistical significance (χ^2^_df(42,1)_ = 1.31, p = 0.106). In contrast, all subjects perform similarly well in the object recognition task even after a 24 hr retention interval, and there was no significant difference between the groups in the likelihood ratio test (χ^2^_df(49,2)_ = 0.715, p = 0.699) (cf. Fig. 5c).

Taken together, these data suggest that WBRT clearly affects hippocampal-dependent memory in the object placement task, and that this deficit worsens with longer retention intervals. In contrast, HSI animals performed similarly to sham-irradiated controls, suggesting that spatial memory is preserved in animals in which the hippocampi were spared from irradiation. Furthermore, varying the training time and retention intervals in the object placement task resulted in a test that is sensitive and robust enough to detect deficits as well as potential improvements in hippocampal-dependent memory.

To show that this result is not dependent on the current preference score pass/fail cutoff value of 53%, we also present the individual data points for each animal in the OP40 and OP70 tests (Supplementary Fig. S1 and Fig. S2). These data show that changing the cutoff value to, for example, 51% or 55% will re-classify only a limited number of pass/fail scores, and the comparisons between the groups remain essentially unchanged.

### HSI results in higher levels of proliferation and neurogenesis compared to WBRT

**Figure 6:**
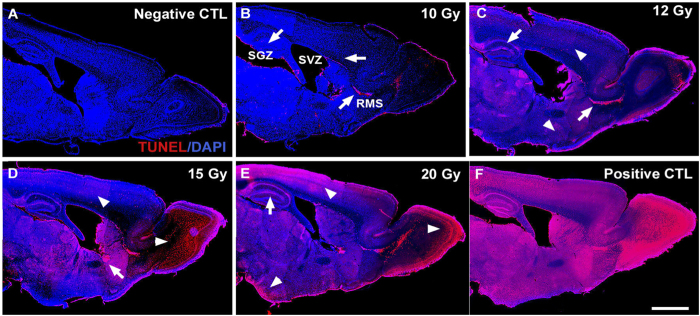
A single dose of 10 Gy results in only limited cell death in the generative zones whereas increasing radiation dose results in widespread cell death. (**A**–**F**) shows photomontages constructed from serial sagittal sections of brains showing the distribution of TUNEL+ apoptotic cells in mouse brains exposed to increasing single fraction doses of WBRT. White arrows point to the apoptotic cells within adult constitutive neurogenesis zones (SGZ: subgranular zone and SVZ: subventricular zone) and RMS: rostral migratory stream. Arrowheads show the distribution of apoptotic cells outside of the generative zones. Scalebar: 1 mm.

We found that a dose of 10 Gy administered in a single fraction, as applied in the behavioral assessment, appears to be appropriate for studying hippocampal-dependent neurocognitive decline, as it resulted in cell death within the subgranular zone (SGZ), subventricular zone (SVZ) and rostral migratory stream (RMS), without additional cell loss in other areas of the brain, cf. Fig. 6.

**Figure 7:**
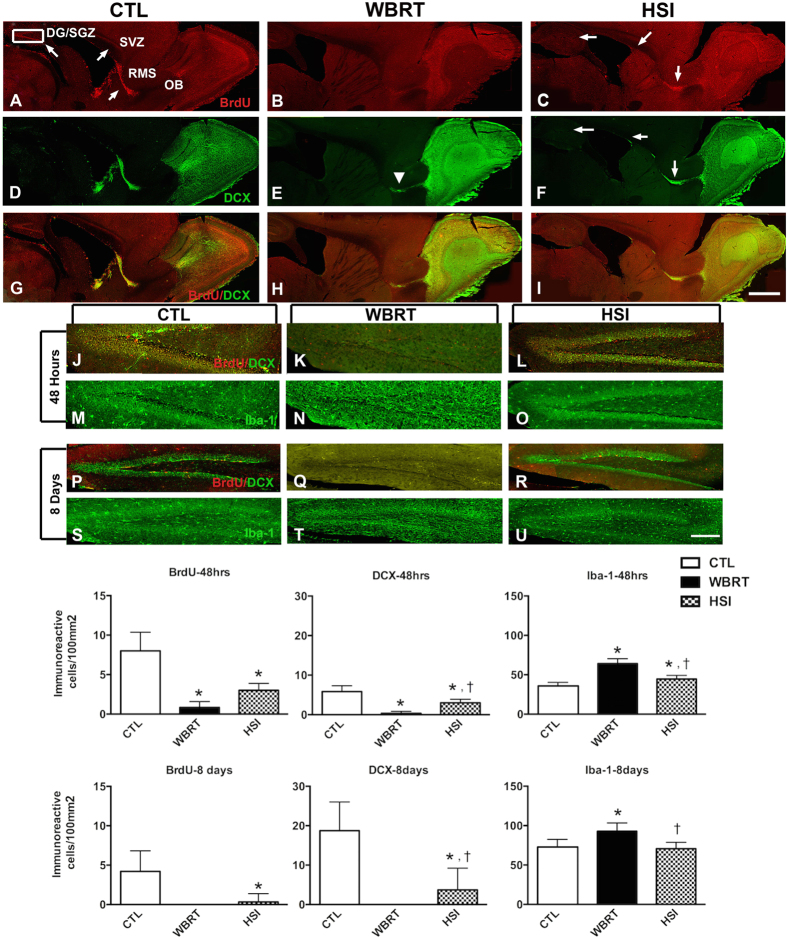
Alterations in NSC-proliferation and NSC-mediated neurogenesis and inflammation in response to WBRT compared to HSI. (**A**–**I**) photomontages constructed from serial sagittal sections of brains showing alterations in the profiles of BrdU+ cells, DCX+ neurons and Iba-1+ microglia 48 hours after cranial irradiation (WBRT vs. HSI) as compared to CTL. White arrows point to the BrdU+ cells within the adult constitutive neurogenesis zone (DG/SGZ: subgranular zone of the dentate gyrus and SVZ: subventricular zone) as well as in the RMS: rostral migratory stream (**A**). WBRT results in almost complete absence of BrdU+ cells and DCX+ young neurons (**B**,**E**) with the exception of a small number of DCX+ neurons within the RMS (**E**-arrowhead). HSI is associated with the maintenance of a small but significant component of these cells (**C**,**F**-white arrows) as compared to CTL (**A**). (**J**–**U**) high-power images corresponding to the inset delineating the DG/SGZ, as shown in image A. There is a significant decrease in number of BrdU+ cells and DCX+ neurons within the SGZ at 48 hours and 8 days after WBRT (**K**,**Q**) as compared to CTL (**J**,**P**). However, HSI preserves significant numbers of BrdU+ cells and DCX+ neurons within the SGZ at 48 hours and 8 days post irradiation (**L**,**R**) as compared to the CTL brains (**J**,**P**). There is also a significant increase in the number of Iba-1+ microglia following WBRT at 48 hours and 8 days after the irradiation (**N**,**T**) whereas HSI can partially decrease the number of these inflammatory cells initially at 48 hours (**O**) is comparable at 8 days (**U**) to CTL (**M**,**S**) within the DG/SGZ. The data are presented as mean ± standard error of the mean where * indicates statistically significant differences compared to CTL and † indicates that HSI is significantly different from WBRT. The number of BrdU+ cells in the DG/SGZ of the WBRT brains 48 hours and 8 days after irradiation as compared to HSI were not significantly different. However, the number of DCX+ cells and Iba-1+ cells in WBRT were found to be significantly different compared to HSI at 48 hours and 8 days. Scalebar: 500 μm (**A**–**I**), 50 μm (**J**–**U**).

As seen in Fig. 7, we observed a complete ablation of BrdU+ proliferating cells and DCX+ neurons within the SGZ, SVZ in the brains examined 48 hours following WBRT at 10 Gy while there was a small number of DCX+ cells in the RMS. There was also a significant number of Iba-1+ microglia within the dentate gyrus and SGZ, indicating clear signs of inflammation. These effects were partially mitigated when animals were treated with HSI with higher levels of proliferating cells and DCX+ neurons. When we repeated the same analyses 8 days after the irradiation procedure, the animals that received HSI continued to have significant number of BrdU+ proliferating cells and DCX+ neurons within the SGZ, but there was no recovery of cell proliferation or neurogenesis in the SGZ of the WBRT group. Finally, while the profile of Iba-1+ microglia in the SGZ of the HSI group was comparable to the number observed in the SGZ of the control group, the number of microglia was significantly higher in the WBRT group. Please note that the one-sided error bars for BrdU and DCX at 8 days are quite large, and as such the lower ends of those error bars are effectively at zero.

As such, the results from immunohistochemical analysis of WBRT- and HSI-treated brains are consistent with the results seen with the behavioral assessment; HSI was shown to rescue hippocampal-dependent cognitive functions when compared to WBRT.

## Discussion

We have successfully demonstrated that our mouse model of hippocampal sparing cranial irradiation can replicate the overall results of the recent RTOG 0933 study, showing that spatial memory function is preserved if the hippocampi are spared from radiation. Our findings demonstrate that there are differences between WBRT irradiated animals and those receiving HSI when assessing hippocampal-dependent tasks but not when examining non-hippocampal specific behavioral tasks. These results were also further confirmed by immunohistochemical analysis in which HSI was shown to result in higher levels of cell proliferation and de novo neurogenesis within the SGZ neural stem cell niche and dentate gyrus, respectively, as well as reduced numbers of microglia, which may be indicative of reduced levels of inflammation.

There was no difference in performance between WBRT and HSI animals in the object recognition task, which is generally non-responsive to manipulations of neural stem cell mediated neurogenesis^25^,^26^. Conversely, we saw a clear difference in the highly hippocampal-dependent object placement task that consistently reflects hippocampal function^27^,^28^,^29^ and is associated with decreased neural stem cell proliferation^25^,^30^. Furthermore, the object placement task is just as effective as the water maze test^31^ but can be repeated for the same subjects (for example, longitudinally), is not confounded by stressors or food deprivation and is similar to tasks of object rotation, pattern recognition and visuospatial memory conducted in humans^32^,^33^,^34^.

Although we did not investigate the mechanisms related to cognitive deficits as a result of hippocampal injury in detail in this study, the results would suggest that intact neural stem cell function and/or proliferation seem to play an important role.

Importantly, preclinical assessment of cognitive function can depend on the species or strain of animal used in the experiments^35^,^36^ as well as the age of animals when irradiated and the time until behavioral assessment. As such, careful consideration must be taken in order to ensure that the appropriate tests and time points are used depending on the animal model of choice.

The model presented here consists of young (16 weeks old) C57BL/6J mice receiving precision cranial irradiation to a single dose of 10 Gy, resulting in spatial memory deficits within two weeks post-irradiation. Since the spatial memory deficit is based on a test in which the animals either pass (show increased interest in a novel object) or fail (no preference shown for the novel object), this could indicate that once hippocampal injury is established a higher number of animals will fail the test compared to controls, but increasing the dose will not cause them to fail with even lower preference scores. This could be tested using the mouse model and behavioral assessment tests presented here, while testing different cranial irradiation doses.

The fact that not all animals receiving WBRT fail the object placement test is consistent with the results found in the randomized trial of motexafin gadolinium whole brain radiation therapy, where 30% of patients treated with WBRT showed a deficit in delayed recall^37^. This would indicate that a dose of 10 Gy in a single fraction used in this mouse model yields results that are translationally relevant to the WBRT dose used in a clinical setting.

Physical shielding of the hippocampus from radiation may sometimes not be possible depending on the intended irradiation target, and it is not the only strategy for mitigating cognitive deficits after cranial irradiation. Other strategies that have been proposed are, for example, subjecting mice to hypoxic conditions following radiation to reduce the detrimental effects of radiation that depend on oxygenation^38^, transplanting embryonic stem cells into the hippocampus two days post WBRT^39^, or reducing the inflammation arising from cranial irradiation^12^.

It is our intention that the presented mouse model, which is able to replicate the results of the RTOG 0933 hippocampal avoidance trial in human patients, will aid researchers in developing new mitigation strategies that are not ethically possible or practically feasible to study in a clinical setting with human subjects.

## Additional Information

**How to cite this article**: Tomé, W. A. *et al*. A mouse model replicating hippocampal sparing cranial irradiation in humans: A tool for identifying new strategies to limit neurocognitive decline. *Sci. Rep*. **5**, 14384; doi: 10.1038/srep14384 (2015).

## Supplementary Material

Supplementary Information
