# Supplementary material for: A mouse model replicating hippocampal sparing cranial irradiation in humans: A tool for identifying new strategies to limit neurocognitive decline
Source: Sci Rep. 2015 Sep 24;5:14384. doi: 10.1038/srep14384 (PMC4585869; doi:10.1038/srep14384)
Supplement: Supplementary Information [file srep14384-s1.doc]

# A mouse model replicating hippocampal sparing cranial irradiation in humans: A tool for identifying new strategies to limit neurocognitive decline

Wolfgang A. Tomé, Ph.D.^1,2*^, Şölen Gökhan, M.D.^3^, N. Patrik Brodin, Ph.D.^1,2^,

Maria E. Gulinello, Ph.D.^4^, John Heard, B.Sc.^2^, Mark F. Mehler, M.D.^3,4,5^,
Chandan Guha, M.D., Ph.D.^1,2^

1 Institute for Onco-Physics, Albert Einstein College of Medicine, Bronx, NY 10461, USA

2 Department of Radiation Oncology, Montefiore Medical Center, Bronx, NY 10461, USA

3 Department of Neurology, Albert Einstein College of Medicine, Bronx, NY 10461, USA

4 Department of Neuroscience, Albert Einstein College of Medicine, Bronx, NY 10461, USA

# 5 Department of Psychiatry and Behavioral Sciences, Albert Einstein College of Medicine, Bronx, NY 10461, USA

# Supplementary material

**Figure S1.** Preference scores plotted as individual data points for the object placement test at 40 min retention. The dashed line shows the pass/fail cutoff of 53%.

**Figure S2.** Preference scores plotted as individual data points for the object placement test at 70 min retention. The dashed line shows the pass/fail cutoff of 53%.
